# High Tobacco Use among Lesbian, Gay, and Bisexual Populations in West Virginian Bars and Community Festivals

**DOI:** 10.3390/ijerph8072758

**Published:** 2011-07-01

**Authors:** Joseph G. L. Lee, Adam O. Goldstein, Leah M. Ranney, Jeff Crist, Anna McCullough

**Affiliations:** 1 Tobacco Prevention and Evaluation Program, Department of Family Medicine, School of Medicine, The University of North Carolina at Chapel Hill, 590 Manning Drive, CB 7595, Chapel Hill, NC 27599, USA; E-Mails: adam_goldstein@med.unc.edu (A.O.G.); leah_ranney@unc.edu (L.M.R.); annamc@unc.edu (A.M.); 2 West Virginia Covenant House, 600 Shrewsbury Street, Charleston, WV 25301, USA; E-Mail: jcrist@wvcovenanthouse.org

**Keywords:** homosexuality, tobacco use, West Virginia

## Abstract

With no information on tobacco use for lesbian, gay, or bisexual (LGB) populations in West Virginia (WV), it is unclear if nationally-identified LGB tobacco disparities also exist in this State. To address this data gap, we conducted a community tobacco survey in bars and events associated with the WV Pride Parade and Festival. Trained community surveyors used electronic and paper survey instruments in bars (n = 6) in three WV cities and community events associated with the WV Pride Parade and Festival. We analyzed results from 386 completed surveys from self-identified LGB individuals. Tobacco use among LGB bar patrons and LGB attendees at Pride-affiliated events was elevated (45%), as was current cigarette use (41%). Users of cigars and chewing tobacco were frequently dual users of cigarettes, with 80% and 60% reporting dual use, respectively. A substantial disparity likely exists in tobacco use among LGB West Virginians. Targeted interventions addressing tobacco use among LGB West Virginians are warranted in these venues, and the addition of a demographic question on sexual orientation would improve data collection and monitoring of this disparity.

## 1. Introduction

The tobacco epidemic has disproportionately affected the health of lesbian, gay, and bisexual (LGB) people and constitutes a major health inequality [1,2]. While little data are available for transgender people, the evidence suggests substantially higher risk of tobacco use [3]. A recent systematic review identified over 47 studies, found generally higher (1.5 to 2.5) odds of smoking among LGB people, and recommended that states begin tracking such data in routine health surveys [2].

The availability of regional and state data on tobacco use prevalence among LGB people remains constrained, however, by the limited number of states that include sexual orientation questions on routine health surveys [4–6]. Virtually no statewide survey data is available from states in the South or Appalachia [2,6,7]. This lack of data hinders state and community interventions as local data are not available to document disparities or to use in program evaluations [4,8]. In the absence of comprehensive state data, the Centers for Disease Control and Prevention (CDC)-funded National LGBT Tobacco Control Network recommends the use of localized convenience samples to examine tobacco use among LGB people [9].

Estimates derived from the American Community Survey and National Survey on Family Growth suggest that over 37,600 LGB individuals live in West Virginia (WV) [10]. WV has one of the highest tobacco use rates in the nation, with 35% of WV residents in the Behavioral Risk Factor Surveillance System (BRFSS) reporting any type of tobacco use [11]. This tobacco use costs the state over 56,100 years of productive life lost annually [12] and represents a major health epidemic [13,14]. No previous research exists on tobacco use among LGBT West Virginians. Only one survey on LGB substance abuse documents high risk of smoking for LGB individuals in one Appalachian state, Kentucky [15].

Tobacco use represents a unique challenge as portions of the tobacco industry’s $34 million per day marketing budget [16] are directed at LGBT communities [17–19]. Industry marketing campaigns are combined with “corporate social responsibility” strategies such as R.J. Reynolds Tobacco Company’s participation in the Corporate Equality Index, a national scoring system for how supportive company policies are of LGBT employees and communities [20]. Researchers have found that tobacco has thus become a normative part of LGB communities [21,22] and ubiquitous in LGB media [23,24]. This is particularly true in bars and clubs, which are a major venue for industry marketing [25–27]. Lesbian women and gay men report more exposure to industry marketing than their straight counterparts [28], further indicating the reach and impact of targeted marketing.

Given the lack of LGB-specific data and the leitmotiv of tobacco industry presence in bars, clubs, and LGB media, Covenant House, a Charleston, WV, interfaith non-profit social services agency, contracted with the UNC Tobacco Prevention and Evaluation Program (TPEP) to: (1) identify if evidence of a tobacco use disparity exists for LGB people in WV; (2) examine interest in quitting among LGB West Virginians who use tobacco; and (3) gauge awareness of existing media outreach to LGB West Virginians run by WV Covenant House.

## 2. Methods

Given the resources available, we determined that random sampling of LGB populations across WV was not feasible. Instead we chose to use venue-based convenience sampling, specifically LGB bars and clubs and the West Virginia Pride Parade and Festival. A venue-based approach is frequently used when resources for population-based sampling are not available and with hard-to-reach populations.

### 2.1. Venue-Selection

To reach a diverse portion of the LGB community, key-informants working with statewide LGB organizations identified bars and clubs frequented by the LGB community. We selected six bars in three cities for their regional diversity and clientele. All six gave permission to conduct surveys. Volunteer surveyors (n = 10) collected surveys between 10 p.m. and 1 a.m. using handheld electronic survey devices. Additionally, staff of the WV Pride Festival and Parade gave permission for surveying at community events, including a volleyball tournament, movie night, and the Pride Parade. Surveyors at community events used a paper questionnaire. Surveyors collected surveys during one night in each city at multiple venues to reduce repeat respondents. Respondents who volunteered they had already been approached were not included a second time. As the survey progressed, the survey team changed the protocol to verbally ask, “Have you taken the West Virginia Tobacco Survey?” when administering the paper survey. All participants provided verbal consent to participate. Signed written consent was not obtained as no identifying information was collected and as the survey provided consent information on the cover page of the paper survey, which instructed respondents not to continue if they did not consent. The electronic survey asked respondents for consent with a yes or no question. Surveys were accompanied by an information sheet providing participants with information on rights, contact information, and the ability to refuse to participate or skip any question. The same language was used on the cover of the paper survey. The University of North Carolina at Chapel Hill Biomedical Institutional Review Board reviewed and approved the research protocol, survey instrument, and consent information (#10-0200).

### 2.2. Survey Instrument

We developed a 16 question survey using standard BRFSS question formats for cigarette smoking (“Have you smoked at least 100 cigarettes in your entire life?” and “Do you now smoke cigarettes every day, some days, or not at all?” [Choice set: every day, some days, not at all]) and quit attempts (“During the past 12 months, have you stopped using tobacco [cigarettes, chew/dip/snuff, or cigars] for one day or longer because you were trying to quit using tobacco?”). We assessed chewing tobacco and cigar use by current use (e.g., “Do you currently smoke cigars every day, some days, or not at all?”). We modified the standard BRFSS age and education questions to have closed response options to facilitate data entry. We ascertained sexual orientation with: “What best describes your sexual orientation? [Choice set: Bisexual, Gay, Lesbian, Straight]”. Because WV is over 94% white [29], we expected to have inadequate data for identifying prevalence by race/ethnicity and thus did not include such a question. The survey instrument screened out non-WV residents and youth under age 18. Electronic surveys employed a skip pattern to eliminate quitting questions for nonusers. The survey asked tobacco users three questions about services: (1) if an LGB-specific quit program would increase respondent confidence in being able to quit (“If you decide to quit, would a program run for gay, lesbian, and bisexual people make you feel more confident about quitting?”); (2) knowledge of Covenant House’s public service announcement (“In the past six months, have you seen a television ad about inviting bad things into your home and choosing to quit smoking?” [the PSA is available from: http://www.wvcovenanthouse.org/health-action-1]); and (3) preferred communication channel to receive information on quitting.

For electronic surveys, we used ArcPad (version 8, ESRI Software, Redlands, California) and handheld Magellan Mobile Mapper 6 units. Depending on noise level and respondent comfort, respondents directly entered data into the device or the surveyor read questions and entered responses.

### 2.3. Survey Implementation

We trained community surveyors on approach, confidentiality, and the avoidance of sampling bias. The surveyor offered each participant a small incentive (rainbow lollipop or organic teabag) and a post-card sized information sheet with a statement about the study, consent information, rights, study contact information, and the eventual web location of the final report. On the other side, the card listed WV Tobacco QuitLine and Covenant House resources. Surveyors collected data during May–June, 2010.

To determine the refusal rate for the survey, surveyors electronically documented refusals during the bar survey through a simple protocol: the first question on consent to participate was answered no by the surveyor when potential respondents declined to participate. We did not capture refusal rates for respondents at the WV Pride Parade and associated activities. We modified the methodology by using paper surveys which were employed to improve efficiency and gain a higher number of respondents. Bar data collection proved slower than desired using electronic surveys.

### 2.4. Analysis

We downloaded data from survey units and into SPSS 17 (IBM, Chicago, Illinois) and entered paper surveys into Excel then combined the data. Two staff members checked 20% of paper surveys for data entry errors. We hypothesized *a priori* that bar surveys would show higher rates of tobacco use and thus that whenever possible the team would stratify data by bar or community event survey. We believed that there would be fewer self-identified straight respondents and given that straight people in LGB bars may not be representative of straight people in general, we did not statistically compare tobacco prevalence between straight and LGB respondents, nor do we make within LGB group comparisons given the sampling limitations of this study.

## 3. Results and Discussion

### 3.1. Survey Participation

We collected 604 surveys in diverse venues across WV. Surveyors worked in six bars and multiple community events, including a dog show, pride parade, volleyball tournament, movie nights, and picnics. Only the bar in Charleston, WV, was covered by a clean indoor air policy, as West Virginia does not have a comprehensive clean indoor air law. In the bar sample, 88% of those approached agreed to participate. Surveyors collected 71% of surveys at festival events and 29% at bars. After excluding non-eligible respondents (straight, under age 18, and/or residing outside of WV [n = 218]), we analyzed results from 386 surveys (Table 1). Sixty-three percent of ineligible respondents were due to self-reported straight identity.

### 3.2. Survey Demographics

Sixty-nine percent of survey participants were male, with relatively similar proportions reported in both the electronic and paper surveys. Respondents were young: 62% reported being under age 40 and only 2% reported being 60 or older (Table 2). Additionally, most respondents reported having completed high school or 2- or 4-year college.

### 3.3. Tobacco Use and Quit Attempts

Tobacco use among respondents in the convenience sample was high (45%), and 41% of respondents reported current cigarette smoking, 4% current smokeless tobacco use, and 11% current cigar use (Table 3). While not comparable to statewide health data due to differences in sampling and the makeup of our sample, these data show a high prevalence of tobacco use among community event participants and bar patrons.

As hypothesized, tobacco use among bar patrons tended to be substantially higher than among participants in the community events. The LGB bar surveys reported higher rates of cigarettes use, smokeless tobacco use, and cigar use. Current smokers in the bar sample were just as likely as those in the festival sample (46% versus 43%) to report quit attempts in the last year.

Lesbian, gay, and bisexual respondents each reported higher use of tobacco than the WV general population: 46% of lesbian women and 38% of gay men reported current smoking. The state rate for WV is 24% for women and 28% for men (Table 4). Respondents reported high levels of cigar use, with between 9% and 27% of LGB respondents using cigars.

### 3.4. Dual Use

LGB cigar smokers and smokeless tobacco users frequently also reported cigarette smoking, with between 60% and 80% reporting such dual use. About half (47%) of smokeless tobacco users reported also smoking cigars (Table 5).

### 3.5. Quit Program Preferences

Among LGB tobacco users interested in quitting, 57% reported that a quit program for LGB people would make them feel more confident about quitting. An additional 23% reported that they did not care, and 20% said that such a program would not increase their confidence. Using a multiple-choice question, we asked how respondents would like to find LGBT-friendly programs to help quitting. The majority of LGB tobacco users interested in quitting suggested Facebook (56%), followed by friends (14%), and WV Pride Web Site (11%).

### 3.6. Awareness of Public Service Announcement Media Campaign

Covenant House aired a WV Tobacco QuitLine promotional public service announcement (PSA) directed at LGB audiences. Survey respondents living in an area where the Covenant House PSA is aired were asked about their awareness of the PSA. Forty-two percent of current smokers surveyed in a Charleston bar reported having seen the PSA.

## 4. Conclusions

This pilot survey found evidence of high rates of tobacco use among LGB West Virginians. The findings of this survey suggest that LGB people living in WV face similar or elevated risk of elevated tobacco use as found in statewide surveys in other states [31,32]. LGB people should be considered for addition to existing [33] WV cancer-related health disparity reduction work. This research suggests that the high burden of tobacco-related disease, including asthma [34], cancer, and other tobaccorelated diseases, constitute a major health disparity for LGB populations.

Why a disparity in tobacco use exists among LGB people remains unclear. However, researchers have posited several explanations. The tobacco industry has clearly targeted LGB individuals with marketing [17]. Stress and discrimination can lead to depression and anxiety, which are a pathway to increased susceptibility to tobacco use [35]. For example, LGB youth in focus groups suggest smoking can make them look tough to protect against harassment [36]. LGB individuals may be less likely to quit due to reduced access to health care [8]. Additionally, bars have long been central LGB community spaces, serving as social hubs and as the nexus of the LGBT rights movements in the 1960s [25,37]. Interviews with bar clients indicate that bars serve as a place to feel accepted [38]. Bars are also places where smoking has been allowed historically. Such consistent exposure to smoking in community spaces may contribute to the disparity, further exacerbating the general lack of comprehensive clean indoor air policies in Appalachia [39].

The survey analysis shows a substantial overlap of multiple tobacco products among cigar smokers and smokeless tobacco users, a problem which has been identified nationally [11]. Our findings of high levels of dual use (60%–80% of cigar and smokeless users also report smoking cigarettes) suggest that current and future interventions could place additional emphasis on addressing dual use.

This survey has a number of limitations. The use of a convenience sample prevents this data from being generalizable to the population of LGB West Virginians who were not surveyed. The results are not generalizable to LGB individuals who do not patronize bars and/or the community events. As bar attendance is associated with increased risk of smoking [40], our reported rates may be higher than the general LGB population. Because we disaggregated gender and sexual orientation, some group sample sizes are small and may thus be unreliable. Caution should be used in interpreting disaggregated prevalence numbers due to size. Further research is needed to verify high cigar use found in our sample, as a previous California-based study reported LGB identity to be associated with lower cigar use and smokeless tobacco use compared to straight identity [41]. Awareness of the Covenant House PSA may have been influenced by question design and social desirability bias. We were unable to measure confirmed awareness. Survey respondents reported being young; as such, this survey does not reflect tobacco use behaviors of older LGB individuals. However, this large sample with a high response rate from a diverse array of community events and bars does provide evidence that the substantial disparity in smoking documented elsewhere may also exist among LGB West Virginians. The evidence is even more compelling viewed in the context of national research that shows a significant disparity exists across time, place, and study design [2]. While our data is not comparable to WV’s general population, we provide such data as a reference point for general thought.

As this convenience sample provides some of the first evidence on tobacco use among LGB West Virginians, it illustrates a clear need for a sexual orientation question in statewide public health surveillance surveys such as the WV BRFSS with its rigorous population sampling. Moreover, this study further documents that the substantial burden of tobacco and its attendant health costs in WV [42] likely extend to and may be higher among LGB communities. The lack of quality data has long hindered LGB health promotion [8], and the importance of such questions in health surveillance is well documented [4,5]. At least 13 other states have collected sexual orientation in the BRFSS [6], often pooling multiple years of data to identify health disparities and design programs [31,32,43].

## Figures and Tables

**Table 1:** Survey participation.

Survey Locations	n
Electronic surveys (including refusals)	
Bar, Charleston	33 (9%)
Three bars, Huntington	35 (9%)
Two bars, Morgantown	35 (9%)
Paper Surveys	
WV Pride Festival (and dog show)	113 (29%)
Power of One Awards Dinner	2 (0.5%)
Huntington Pride Picnic	35 (9%)
Pride Wine Tasting	13 (3%)
Mr./Ms. Pride Competition	52 (14%)
Bear Contest	16 (4%)
Young adult Monte Carlo night	7 (2%)
Pride film night	6 (2%)
Pride volleyball	14 (4%)
Pride Social	25 (7%)

**Total**	**386**

Note: Percentage totals do not add to 100 due to rounding.

**Table 2:** Demographic characteristics of survey sample by sexual orientation.

Sexual Orientation					
	Lesbian (n = 89)	Gay (n = 245)	Bisexual Women (n = 28)	Bisexual Men (n = 24)	Total (n = 386)
**Age**					
18–24	20%	20%	32%	21%	21%
25–29	16%	14%	29%	8%	15%
30–34	18%	14%	7%	17%	15%
35–39	8%	11%	14%	21%	11%
40–44	8%	14%	7%	13%	12%
45–49	12%	11%	7%	13%	11%
50–54	12%	8%	–	4%	8%
55–59	6%	5%	4%	4%	5%
60+	–	3%	–	–	2%

**Highest Education**					
Some high school	2%	2%	-	-	2%
High school	32%	24%	29%	33%	27%
2 year college	35%	25%	25%	29%	27%
4 year college	19%	33%	29%	33%	30%
Graduate school	12%	16%	18%	4%	14%

**Survey Mode**					
Electronic (bars)	15%	26%	54%	46%	27%
Paper (festival)	85%	74%	46%	54%	73%

**Table 3:** LGB tobacco use and quit attempts by sexual orientation among WV LGB.

	LGB, bar sample (n = 103)	LGB, festival sample (n = 283)	LBG, overall (n = 386)	WV General Population [30]
Current tobacco user	57%	40%	45%	35% (2008 BRFSS) [11]
Current smoker	51%	38%	41%	26% (2009 BRFSS)
Current smokeless tobacco user	8%	2%	4%	6% (2006–2007 TUS-CPS)
Current cigar smoker	21%	7%	11%	3% (2006–2007 TUS-CPS)
Quit attempts in last year (current smokers)	46%	43%	44%	50% (2009 BRFSS)

**Table 4:** LGB tobacco use behaviors and quit attempts by sexual orientation.

Sexual Orientation					WV General Population [30]	
	Lesbian (n = 89)	Gay (n = 245)	Bisexual Woman (n = 28)	Bisexual Man (n = 24)	Women	Men
Current tobacco user	49%	40%	62%	54%	Aggregated: 35% (2008 BRFSS) [11]	
Current smoker	46%	38%	48%	46%	24% (2009 BRFSS)	28% (2009 BRFSS)
Current smokeless tobacco user	3%	3%	4%	17%	0.3% (2006–2007 TUS-CPS)	12% (2006–2007 TUS-CPS)
Current cigar smoker	12%	9%	27%	13%	0.5% (2006–2007 TUS-CPS)	5.0 (2006–2007 TUS-CPS)
Quit attempts in last year (current smokers)	33%	52%	42%	27%	49% (2009 BRFSS)	51% (2009 BRFSS)

Note: TUS-CPS = Tobacco Use Supplement to the Current Population Survey.

**Table 5:** Dual use of tobacco products among WV LGB tobacco users.

	% Using cigarettes	% Using cigars	% Using smokeless
Cigarette smokers	–	22%	6%
Cigar smokers	80%	–	16%
Smokeless users	60%	47%	–

Note: cigar smokers and smokeless users are defined as using “some days” or “every day.”

## References

[1] Gruskin EP, Greenwood GL, Matevia M, Pollack LM, Bye LL (2007). Disparities in smoking between the lesbian, gay, and bisexual population and the general population in California. Am. J. Public Health.

[2] Lee JG, Griffin GK, Melvin CL (2009). Tobacco use among sexual minorities in the USA, 1987 to May 2007: A systematic review. Tob. Control.

[3] Sanchez NF, Sanchez JP, Danoff A (2009). Health care utilization, barriers to care, and hormone usage among male-to-female transgender persons in New York City. Am. J. Public Health.

[4] Sell RL, Becker JB (2001). Sexual orientation data collection and progress toward Healthy People 2010. Am. J. Public Health.

[5] Sell RL, Dunn PM (2008). Inclusion of lesbian, gay, bisexual and transgender people in tobacco use-related surveillance and epidemiological research. J. LGBT Health Res.

[6] Lee JG (2009). Social ecology of tobacco surveillance data for sexual and gender minority populations. Nicotine Tob. Res.

[7] Ryan H, Wortley PM, Easton A, Pederson L, Greenwood G (2001). Smoking among lesbians, gays, and bisexuals: a review of the literature. Am. J. Prev. Med.

[8] Mayer KH, Bradford JB, Makadon HJ, Stall R, Goldhammer H, Landers S (2008). Sexual and gender minority health: what we know and what needs to be done. Am. J. Public Health.

[9] NatNet Promising practices for comprehensive tobacco control programs: Identifying and eliminating LGBT disparities.

[10] Gates G (2006). Same-Sex Couples and the Gay, Lesbian, Bisexual Population: New Estimates from the American Community Survey.

[11] CDC (2010). Any tobacco use in 13 States—behavioral risk factor surveillance system 2008. MMWR.

[12] CDC (2009). State-specific smoking-attributable mortality and years of potential life lost—United States, 2000–2004. MMWR.

[13] Adkins BW (2006). There is no greater health epidemic in West Virginia than tobacco addiction!. WV Med. J.

[14] Behringer B, Friedell GH (2006). Appalachia: Where place matters in health. Prev. Chronic. Dis.

[15] Skinner WF, Otis MD (1996). Drug and alcohol use among lesbian and gay people in a southern U.S. sample: Epidemiological, comparative, and methodological findings from the Trilogy Project. J. Homosex.

[16] FTC (2009). Cigarette Report for 2006.

[17] Stevens P, Carlson LM, Hinman JM (2004). An analysis of tobacco industry marketing to lesbian, gay, bisexual, and transgender (LGBT) populations: Strategies for mainstream tobacco control and prevention. Health Promot. Pract.

[18] Washington HA (2002). Burning Love: Big tobacco takes aim at LGBT youths. Am. J. Public Health.

[19] Smith EA, Malone RE (2003). The outing of Philip Morris: Advertising tobacco to gay men. Am. J. Public Health.

[20] Comer M (2009). Stamp of approval: Gay researcher questions Human Rights Campaign’s perfect rating of Reynolds Tobacco Co. Q-Notes.

[21] Offen N, Smith EA, Malone RE (2008). Is tobacco a gay issue? Interviews with leaders of the lesbian, gay, bisexual and transgender community. Cult. Health Sex.

[22] Smith EA, Thomson K, Offen N, Malone RE (2008). “If you know you exist, it’s just marketing poison”: Meanings of tobacco industry targeting in the lesbian, gay, bisexual, and transgender community. Am. J. Public Health.

[23] Smith EA, Offen N, Malone RE (2005). What makes an ad a cigarette ad? Commercial tobacco imagery in the lesbian, gay, and bisexual press. J. Epidemiol. Community Health.

[24] Smith EA, Offen N, Malone RE (2006). Pictures worth a thousand words: Noncommercial tobacco content in the lesbian, gay, and bisexual press. J. Health Commun.

[25] Leibel K, Lee JG, Goldstein AO, Ranney LM (2011). Barring Intervention? Lesbian and Gay Bars as an Underutilized Venue for Tobacco Interventions. Nicotine Tob Res.

[26] Gilpin EA, White VM, Pierce JP (2005). How effective are tobacco industry bar and club marketing efforts in reaching young adults?. Tob. Control.

[27] Katz SK, Lavack AM (2002). Tobacco related bar promotions: insights from tobacco industry documents. Tob. Control.

[28] Dilley JA, Spigner C, Boysun MJ, Dent CW, Pizacani BA (2008). Does tobacco industry marketing excessively impact lesbian, gay and bisexual communities?. Tob. Control.

[29] USCB 2005–2009 American Community Survey 5-Year Estimates: Data Profile Highlights—West Virginia.

[30] CDC State Tobacco Activities Tracking and Evaluation (STATE) System.

[31] Dilley JA, Simmons KW, Boysun MJ, Pizacani BA, Stark MJ (2010). Demonstrating the importance and feasibility of including sexual orientation in public health surveys: Health disparities in the Pacific Northwest. Am. J. Public Health.

[32] Conron KJ, Mimiaga MJ, Landers SJ (2010). A population-based study of sexual orientation identity and gender differences in adult health. Am. J. Public Health.

[33] Brown PK, Heady HR, Gainor SJ, Harrison D, Daven M (2009). Perspectives on cancer health disparities in West Virginia. WV Med. J.

[34] Blosnich J, Jarrett T, Horn K (2010). Disparities in smoking and acute respiratory illnesses among sexual minority young adults. Lung.

[35] Meyer IH (2003). Prejudice, social stress, and mental health in lesbian, gay, and bisexual populations: conceptual issues and research evidence. Psychol. Bull.

[36] Remafedi G (2007). Lesbian, gay, bisexual, and transgender youths: who smokes, and why?. Nicotine Tob. Res.

[37] Howard J (1999). Men Like That: A Southern Queer History.

[38] Gruskin E, Byrne K, Kools S, Altschuler A (2006). Consequences of frequenting the lesbian bar. Women Health.

[39] Ferketich AK, Liber A, Pennell M, Nealy D, Hammer J, Berman M (2010). Clean indoor air ordinance coverage in the Appalachian region of the United States. Am. J. Public Health.

[40] Trocki KF, Drabble LA, Midanik LT (2009). Tobacco, marijuana, and sensation seeking: Comparisons across gay, lesbian, bisexual, and heterosexual groups. Psychol. Addict. Behav.

[41] Gruskin EP, Greenwood GL, Matevia M, Pollack LM, Bye LL, Albright V (2007). Cigar and smokeless tobacco use in the lesbian, gay, and bisexual population. Nicotine Tob. Res.

[42] Lengerich EJ, Tucker TC, Powell RK, Colsher P, Lehman E, Ward AJ, Siedlecki JC, Wyatt SW (2005). Cancer incidence in Kentucky, Pennsylvania, and West Virginia: disparities in Appalachia. J. Rural Health.

[43] VanKim NA, Padilla JL, Lee JG, Goldstein AO (2010). Adding sexual orientation questions to statewide public health surveillance: New Mexico’s experience. Am. J. Public Health.

